# Large Draining Focal Fibrous Hyperplasia Secondary to Periapical Granuloma

**DOI:** 10.1155/2018/4850901

**Published:** 2018-06-03

**Authors:** E. I. Ogbureke, M. A. Couey, N. Vigneswaran, C. D. Johnson

**Affiliations:** ^1^Department of General Practice and Dental Public Health, School of Dentistry, The University of Texas Health Science Center at Houston, Houston, TX, USA; ^2^Department of Oral and Maxillofacial Surgery, School of Dentistry, The University of Texas Health Science Center at Houston, Houston, TX, USA; ^3^Department of Diagnostic & Biomedical Science, School of Dentistry, The University of Texas Health Science Center at Houston, Houston, TX, USA

## Abstract

Periapical granuloma is a pathological diagnosis associated clinically and radiographically with a nonvital tooth and a periapical radiolucency, respectively. It is frequently seen as a sequela of long-standing pulpal necrosis. Often times, a draining fistula is observed near the nonvital tooth. We report an unusual case of a large draining focal fibrous hyperplasia in association with a large periapical granuloma treated at our clinic. The diagnosis was made by the clinical presentation, radiologic and histopathologic findings.

## 1. Introduction

Periapical granuloma, also referred to as chronic apical periodontitis, is a defensive reaction in response to bacterial infection within the pulp chamber which spreads to the root apex [1]. It is a long-standing inflammation of the periodontium that is characterized by the presence of a granulomatous tissue [2]. Radiographically, periapical granulomas are generally indistinguishable from periapical abscesses or cysts. They are often asymptomatic unless the infection spreads to the surrounding tissues.

## 2. Case Report

A 59-year-old man presented to the urgent care clinic at the School of Dentistry complaining of an upper lip mass for one-year duration. The mass started out as a small bump and had grown steadily since then. 3 months prior to his presentation, a draining parulis developed on the mass. The patient had no history of systemic symptoms such as fever, chills, weight loss, or fatigue. He was aware of a dark-colored “dead tooth” for several decades in the area of concern but denied any previous history of swelling in the area. The patient had recently moved to the United States from Nigeria and had previously been without access to adequate dental care. The patient said that a doctor in Nigeria told him that the lesion was likely cancerous.

**Figure 1:**
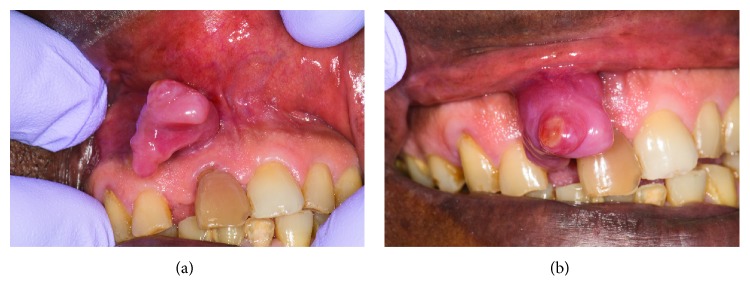
(a) Picture of soft tissue exophytic lesion in buccal vestibule. (b) Picture of soft tissue lesion showing purulent exudate.

On exam, there was a large, painless, fibrous, exophytic mass in the anterior maxillary labial vestibule (Figures 1(a) and 1(b)). The base of the mass approximated the apex of tooth #8. A yellow purulent material was observed draining from the parulis (Figure 1(b)). Tooth #8 was discolored and was confirmed to be nonvital on pulp testing. There was a significant gap between teeth #7 and 8. Tooth #8 was displaced medially and was extruded relative to the adjacent dentition.

**Figure 2:**
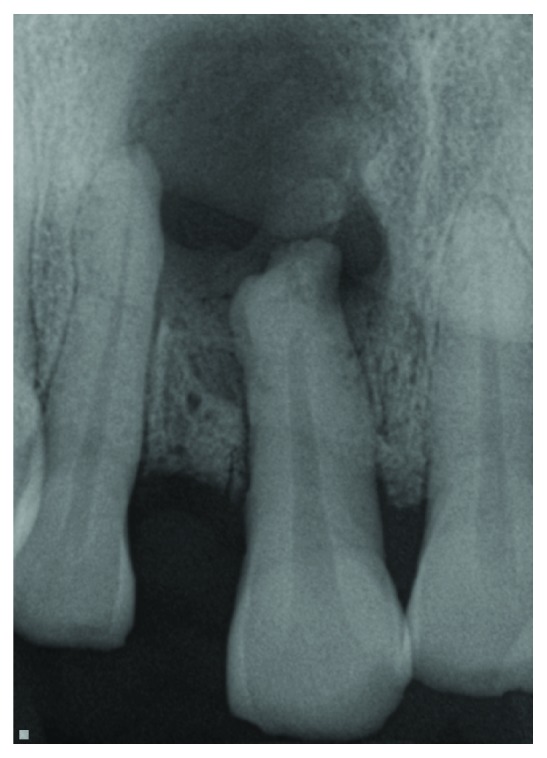
Periapical radiograph of large radiolucency associated with necrotic tooth #8.

**Figure 3:**
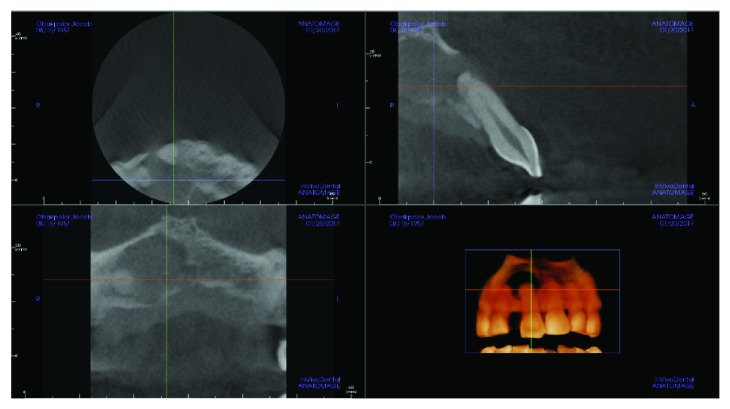
CBCT image showing large cystic-appearing defect in the anterior maxilla with perforation of the buccal and palatal cortices.

A periapical radiograph revealed a large unilocular radiolucency associated with the apex of tooth #8 (Figure 2). Cone-beam computed tomography again demonstrated a large cystic-appearing defect in the anterior maxilla with perforation of the buccal and palatal cortices Figure 3. The lesion extended to the nasal floor on the ipsilateral side.

The patient was referred to the oral surgery department for excisional biopsy. After tooth #8 was removed, an incision was made around the base of the stalk that connected the mass to the labial and alveolar mucosa. Sharp dissection was used to free the mass, and the specimen was sent for histopathologic analysis. The mass communicated with a cystic lesion of the maxilla. The cyst was enucleated with a curette and also sent for pathology. Perforation of the cyst through the buccal and palatal cortices was noted during the procedure. Slight undermining of the wound margins allowed for closure with resorbable sutures.

**Figure 4:**
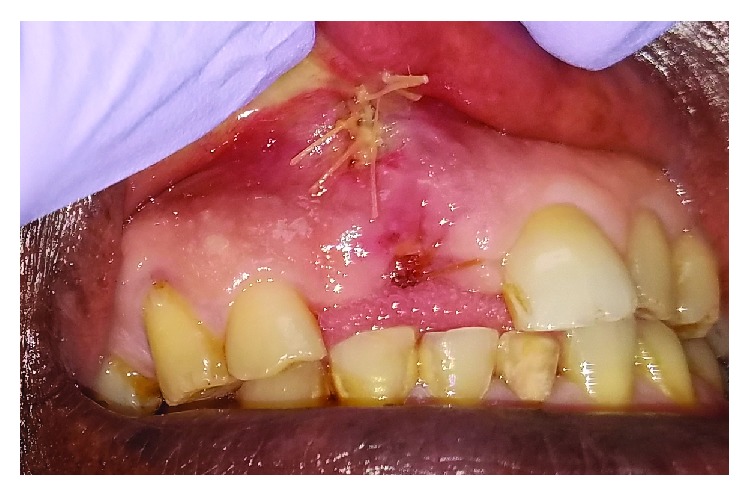
Picture at one-week follow-up.

At the patient's one-week follow-up (Figure 4), he was doing very well. He reported minimal pain, no neurosensory disturbances, and no systemic or local symptoms of infection. He and his family were very relieved to learn that the lesion was benign. He was happy with his appearance after having the mass removed.

## 3. Discussion

A search on PUBMED, google scholar, and google using the terms “focal fibrous hyperplasia” and “draining fibrous hyperplasia” yielded no reported cases of a similar occurrence in the English literature. Focal fibrous hyperplasia (FFH), is a reactive, inflammatory hyperplastic lesion of the connective tissue [3]. It is presumed that mechanical trauma is the primary cause hence it more commonly occurs in the buccal mucosa and tongue [3]. FFH like other reactive soft tissue oral lesions such as pyogenic granuloma (PG), peripheral giant cell granuloma (PGCG), and peripheral ossifying fibroma (POF) are more common in females, and hormones are thought to play a part in its etiology [3, 4]. FFH is distinguishable from the aforementioned lesions by histology. Generally, the teeth associated with FFH and the lesions of PG, PGCG, and POF are vital. The patient in this report is male, and tooth #8 is nonvital and shows a large periapical radiolucency (Figure 2). This leads the authors to postulate that pulpal necrosis of #8 and the subsequent periapical pathology led to a fistula formation in the buccal vestibule adjacent to #8. Trauma and irritation of the fistula opening may have initiated the hyperplastic lesion of FFH.

The location of the periapical lesion is consistent with studies that confirm the anterior maxilla as being the commonest site for periapical granulomas and cysts [4]. This is probably because the anterior maxilla is more prone to trauma than other tooth-bearing areas of the jaws.

## 4. Histopathological Findings

On gross examination, the growth excised from the gingiva consisted of a yellowish-tan, irregular-shaped fragment of soft tissue measuring 1.5 × 1.2 × 0.9 cm. The intraosseous lesions curated from the cystic lesion of the anterior maxilla composed of multiple tan and irregular-shaped fragments of soft tissue measuring in aggregate 2 × 2 × 0.6 cm.

**Figure 5:**
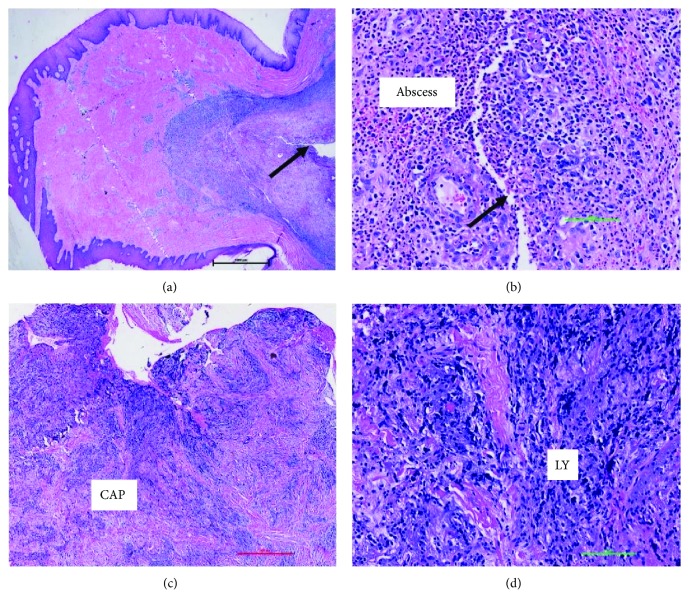
Microscopic findings of the gingival growth (a and b) and periapical radiolucent lesion associated with tooth #8 (c and d). Fibrous growth in the gingiva exhibits opening of a sinus tract (arrows) surrounded by an abscess. Periapical radiolucent lesion revealed chronic apical periodontitis (CAP) consisting of mostly lymphocytic infiltrate (LY).

Microscopic examination of the gingival growth revealed a soft tissue mass surfaced by parakeratinized hyperplastic stratified squamous epithelium (Figure 5). The underlying lamina propria exhibited area of dense fibrous hyperplasia with chronic inflammatory cell infiltrate with increased vascularity. Underneath the hyperplastic, fibrous connective tissue is an abscess consisting of granulation tissue with sheets of neutrophils intermixed with histiocytes and necrotic cellular debris (Figure 5). A sinus tract was present within the middle of the abscess. Biopsy curetted from the accompanying intraosseous lesion revealed dense fibrous connective tissue and granulation tissue with mixed inflammatory cell infiltrate (Figure 5).

## 5. Conclusion

Few oral lesions are unique or distinctly remarkable in nature. Therefore, it is imperative for oral healthcare providers to follow well-established matrices when encountering atypical oral presentations. The literature strongly suggests high reliability of patients' self-reporting and a thorough account including medical history and the history of the lesion as essential to the diagnostic process. Of secondary importance is delineation of the physical characteristics of the lesion including the size, shape, color, and texture. Radiographic evaluation may assist in narrowing the differential. In our case, despite the atypical appearance, a presumptive differential was limited by the history of the lesion to a fibrous hyperplasia. We, however, advise vigilance in the need for histopathologic evaluation and proper referral when required.
